# Retraction: Knockdown of Gab1 Inhibits Cellular Proliferation, Migration, and Invasion in Human Oral Squamous Carcinoma Cells

**DOI:** 10.32604/or.2024.055039

**Published:** 2024-07-17

**Authors:** Oncology Research Editorial Office

The published article titled “Knockdown of Gab1 Inhibits Cellular Proliferation, Migration, and Invasion in Human Oral Squamous Carcinoma Cells” has been retracted from *Oncology Research*, Vol. 26, No. 4, 2018, pp. 617–624.

DOI: 10.3727/096504017X15043589260618

URL: https://www.techscience.com/or/v26n4/56674

Following the publication, concerns have been raised about a number of figures in this article. The Western Blot data shown in Figs. 2a, 2b were strikingly similar to data appearing in different form in other articles written by different authors at different research institutes that had already been published.

The authors were contacted and invited to comment on the concerns raised and to provide the original, unmodified figures, but did not respond. The Editors-in-Chief therefore no longer have confidence in the integrity of the data in this article and decided to retract this article.

All authors have not responded to correspondence about this retraction. As a responsible publisher, we hold the reliability and integrity of our published content in high regard. We deeply regret any inconvenience caused by this situation to our readers and all concerned parties.

